# Effects of Respiratory Vaccines in Older Adults with Cardiovascular Diseases: A Scoping Review

**DOI:** 10.3390/vaccines14040308

**Published:** 2026-03-29

**Authors:** Fernando M. Runzer-Colmenares, Nelson Luis Cahuapaza-Gutierrez, Cielo Cinthya Calderon-Hernandez, Mariam Miyanay Umeres-Bravo

**Affiliations:** 1 CHANGE Research Working Group, Universidad Científica del Sur, Lima 15067, Peru; 100065659@cientifica.edu.pe (N.L.C.-G.); 100052073@cientifica.edu.pe (C.C.C.-H.);; 2 Facultad de Ciencias de la Salud, Carrera de Medicina, Universidad de Aquino Bolivia, Cochabamba, Bolivia

**Keywords:** vaccines, respiratory vaccines, cardiovascular disease, older adults, scoping review (source: MeSH NLM)

## Abstract

**Background/Objectives**: Vaccination against respiratory viruses—such as respiratory syncytial virus (RSV), pneumococcal disease, influenza, and COVID-19—may reduce the risk of adverse outcomes in older adults with cardiovascular disease. This study conducted a scoping review of the effects of respiratory vaccines in older adults with cardiovascular disease. **Methods**: We included studies evaluating adults aged ≥ 60 years with cardiovascular disease who received different types of respiratory vaccines. Eligible designs comprised clinical trials, observational cohort studies, and other relevant studies. Editorials, commentaries, and non-original publications were excluded. A comprehensive and targeted literature search was conducted in PubMed, Scopus, EMBASE, and Web of Science from database inception through January 2026. **Results**: A total of 25 studies were included, encompassing 1,782,787 adults aged ≥ 60 years with cardiovascular disease who received various respiratory vaccines. RSV vaccines were associated with a lower incidence of cardiorespiratory hospitalization and stroke among vaccinated individuals. Pneumococcal vaccines showed that sequential dual vaccination strategies were associated with a lower risk of cardiovascular events. Influenza vaccination was associated with improved cardiovascular outcomes, lower mortality, and reduced adverse events. COVID-19 vaccines were associated with reductions in mortality and hospitalizations. These benefits are particularly relevant in an older population with a high burden of comorbidities; therefore, complete vaccination schedules, including booster doses, should be considered a central strategy for prevention and comprehensive management in this high-risk group. **Conclusions**: Vaccination against respiratory viruses in older adults with cardiovascular disease demonstrates an overall favorable/acceptable profile of efficacy and safety, with reductions in mortality, hospitalizations, and cardiovascular events, without a significant increase in serious adverse events.

## 1. Introduction

Cardiovascular diseases (CVD) are the leading cause of morbidity and mortality worldwide, with a disproportionately greater burden among older adults [1,2]. Aging is associated with profound remodeling of the immune system, characterized by immunosenescence and a chronic low-grade inflammatory state, which directly contributes to increased susceptibility to infections and to the severity of their complications in older adults [3,4]. In this context, acute respiratory infections represent an important trigger for cardiovascular events such as acute myocardial infarction, stroke, acute heart failure, and venous thromboembolism [5,6,7].

Respiratory pathogens including influenza virus, respiratory syncytial virus (RSV), *Streptococcus pneumoniae*, and severe acute respiratory syndrome coronavirus 2 (SARS-CoV-2)—are highly prevalent in the older adult population and are associated with substantial cardiovascular morbidity [7,8]. Infections caused by these pathogens may exacerbate CVD by inducing systemic inflammation, endothelial dysfunction, and a prothrombotic state, thereby promoting ischemia, thrombosis, and destabilization of atherosclerotic plaques. In addition, hypoxemia, sympathetic activation, and, in some cases, direct myocardial inflammation or infection may precipitate arrhythmias, heart failure, and acute cardiovascular events, particularly in patients with pre-existing disease [9,10].

Vaccination against respiratory pathogens has been widely implemented as a primary preventive strategy to reduce exacerbations, morbidity, and mortality associated with infections [11]. Current evidence also suggests that vaccines such as those for influenza, pneumococcus, RSV, and COVID-19 may confer additional cardiovascular benefits [12]. Several studies have reported reductions in cardiovascular events, hospitalizations, and all-cause mortality following vaccination; however, the magnitude and consistency of these effects vary according to vaccine type, platform, and the underlying cardiovascular condition [12,13,14,15,16]. Furthermore, standardization is lacking, and the impact of these vaccines in adult populations with cardiovascular disease remains under investigation.

Therefore, the present study conducted a scoping review to systematically map the available research on the efficacy, safety, and immunogenicity of respiratory vaccines in older adults with cardiovascular disease.

## 2. Materials and Methods

### 2.1. Protocol and Registration

The study protocol was developed in accordance with the Preferred Reporting Items for Systematic Reviews and Meta-Analyses Protocols (PRISMA-P 2015) guidelines [17]. The scoping review protocol was registered in the Open Science Framework database on 11 January 2026, and is available at (https://osf.io/hrymx/files/fyevr, accessed on 11 January 2026). This scoping review was conducted and reported in accordance with the PRISMA-ScR statement [18].

### 2.2. Eligibility Criteria

Eligibility criteria were based on the predefined PICO research question framework: P (population), I (intervention), C (comparator), and O (outcomes). The population included adults aged ≥ 60 years with cardiovascular diseases such as heart failure, coronary atherosclerosis, myocardial infarction, stroke, and atrial fibrillation. Interventions comprised all respiratory vaccines against influenza, respiratory syncytial virus, pneumococcal disease, and COVID-19, regardless of dose (first, second, third, and booster) and vaccine platform or technology. Comparators included placebo or control groups. Outcomes of interest were efficacy/effectiveness (measured by hospitalizations and all-cause mortality), safety (adverse events and/or side effects), and immunogenicity (humoral and cellular immune responses). Eligible study designs included randomized controlled trials, observational cohort studies (population-based or hospital-based), nested case–control studies, and secondary analyses.

Studies were excluded if they included: (i) populations younger than 60 years, including children and adolescents; (ii) older adults without cardiovascular disease and/or healthy individuals; or (iii) other types of vaccines or interventions. We also excluded letters to the editor, editorials, clinical images, comments, notes, correspondence, conference abstracts, reports, narrative reviews, systematic reviews, meta-analyses, in vivo and in vitro studies, books, book chapters, journalistic articles, and opinion pieces. Studies not published in English or without full-text availability were also excluded.

### 2.3. Information Sources

The preliminary search was conducted on 1 October 2025, and updated on 15 December 2025. The final search presented in this study was conducted on 11 February 2026. Electronic databases searched included PubMed, Scopus, and EMBASE, as well as the Web of Science platform. Gray literature was also explored through Google Scholar. In addition, reference lists of included studies were manually screened to ensure comprehensive coverage.

### 2.4. Search Strategy

A comprehensive search strategy was developed for each database using MeSH terms from the National Library of Medicine (NLM) and database-specific commands such as TIAB, MeSH, and (TS=). The search terms included “Older adults,” “Aging,” “Elderly,” “Influenza Vaccines,” “Respiratory Syncytial Virus Vaccines,” “Pneumococcal Vaccines,” and “COVID-19 Vaccines,” combined using Boolean operators (AND, OR). The search was limited to English-language publications and to studies published between 2016 and 2026 to capture contemporary evidence. The full search strategies for each database are detailed in Supplementary Material S1.

### 2.5. Process for Selection of Sources of Evidence

All retrieved records were imported into EndNote to remove duplicates and subsequently exported to the Rayyan QCRI web platform for screening. Two reviewers (NLCG and CCCH) independently screened titles and abstracts of potentially eligible studies. Full-text articles were then assessed for eligibility. Disagreements were resolved by consensus.

### 2.6. Data Charting Process

Two reviewers (NLCG and CCCH) developed a standardized data extraction form to collect key study characteristics, including first author, year of publication, country, study design, total population, age group, sex, cardiovascular condition, inclusion and exclusion criteria, vaccine type, number of doses, vaccine platform/technology, outcomes, and conclusions. Discrepancies were resolved by consensus, and when necessary, a third reviewer (FRMC) was consulted.

### 2.7. Data Items

Data were collected and synthesized on general study characteristics, including first author, year of publication, and study design. Population characteristics and contextual factors were also extracted, including age, sex, clinical history, risk factors, type of cardiovascular disease, and population size or setting. Regarding interventions, information on vaccine type, vaccine platform or technology, number of doses, and evaluated outcomes—specifically efficacy/effectiveness, safety, and immunogenicity—was collected.

### 2.8. Synthesis of Results

Quantitative descriptive measures, including frequencies (n) and percentages (%), were used for data analysis and synthesis, along with a qualitative synthesis of the available evidence. Data management and analysis were performed using STATANow, version 19 SE. A PRISMA 2020 flow diagram was used to transparently describe the study selection process. In accordance with PRISMA-ScR methodological guidance for scoping reviews, risk of bias assessment, study quality appraisal, and subgroup or sensitivity analyses were not conducted, as these procedures are not applicable to this type of review design.

## 3. Results

### 3.1. Selection of Sources of Evidence

**Figure 1:**
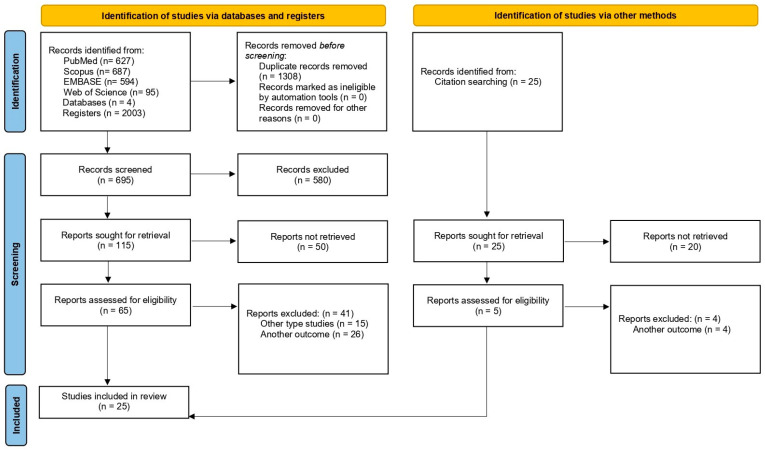
Selection of studies on respiratory vaccines in older adults with cardiovascular disease. Flow diagram “Preferred Reporting Items for Systematic Reviews and Meta-Analyses” PRISMA 2020.

Systematic bibliographic searches were conducted in the previously described databases. A total of 2003 records were identified; after the removal of duplicates, 695 manuscripts were included in the screening phase. Through title and abstract review, 115 studies were selected for eligibility assessment, while 580 were excluded for not meeting the predefined protocol criteria. Full-text evaluation led to the exclusion of 50 articles, primarily due to the lack of reporting of the outcomes of interest initially identified in the abstracts. Subsequently, the remaining 65 manuscripts were analyzed in detail, of which 41 studies were excluded for not meeting the established inclusion criteria. In addition, the reference lists of the included studies were manually screened, and one additional relevant study was incorporated. Ultimately, a total of 25 studies were included in the review [19,20,21,22,23,24,25,26,27,28,29,30,31,32,33,34,35,36,37,38,39,40,41,42,43]. Figure 1 presents the study selection process for the scoping review.

### 3.2. Respiratory Syncytial Virus Vaccines

For the qualitative analysis of respiratory syncytial virus vaccines in patients with cardiovascular disease, two studies were included, both corresponding to prespecified secondary analyses of clinical trials published in 2025 and analyzed over follow-up periods spanning 2024–2025. Both studies were based on the Danish DAN-RSV network and included a total of 80,019 patients who received RSV vaccination, with a mean age above 70 years.

The study by Lassen et al. [19] evaluated the effectiveness of the bivalent prefusion F protein-based RSV vaccine (RSVpreF) in adults aged ≥ 60 years compared with unvaccinated individuals. The results demonstrated a lower incidence of all-cause cardiorespiratory hospitalization in the vaccinated group, with an absolute rate reduction of 2.90 and a vaccine effectiveness of 9.9%.

Similarly, the study by Pareek et al. [20] assessed the effectiveness of the same bivalent vaccine in adults aged ≥ 60 years with atherosclerotic cardiovascular disease. A lower incidence of stroke was observed in the RSVpreF group compared with the control group; however, the effectiveness was similar between groups for most of the evaluated outcomes. Table 1 and Table 2 present in detail the methodological characteristics and main results of the included studies.

### 3.3. Pneumococcal Vaccines

For the qualitative analysis of the 23-valent pneumococcal polysaccharide vaccine (PPSV23) and the 13-valent pneumococcal conjugate vaccine (PCV13) in older adults with cardiovascular disease, a single study was identified, published in 2024 with a follow-up period from 2012 to 2020.

The study by Tong et al. [21], a retrospective cohort conducted in Hong Kong, evaluated the protective effect of a sequential pneumococcal vaccination strategy compared with single-vaccine administration in 262,421 older adults with cardiovascular disease (single dose: 157,244; sequential vaccination: 72,875). The findings demonstrated that dual sequential vaccination was associated with a lower risk of cardiovascular events compared with either PCV13 alone or PPSV23 alone. These results suggest that a sequential strategy may provide additional benefits in reducing cardiovascular risk and should be considered in clinical decision-making regarding pneumococcal vaccination in this high-risk population.

### 3.4. Influenza Vaccines

For the qualitative analysis of influenza vaccines in older adults with cardiovascular disease, 18 studies were included, encompassing cohort designs, cohort analyses, case–control studies, self-controlled case series, secondary analyses of trials, and trial emulations. The publication period ranged from 2016 to 2025, with follow-up intervals spanning from 1990 to 2022. Most studies were conducted in Taiwan, followed by China, the United States, and Spain. Overall, 1,501,735 vaccinated older adults (≥60 years) were included.

The study by Hsu et al. [22] evaluated the effect of influenza vaccination on reducing the risk of infarction in older adults, showing that the risk of myocardial infarction may decrease—particularly in men—when there is a good match between the vaccine and circulating strains in individuals older than 65 years. Chiang et al. [23] demonstrated that influenza vaccination is associated with a reduction in major adverse cardiovascular events, myocardial infarction, and stroke. Liu et al. [24] reported dose–response and synergistic protective effects against hemorrhagic stroke in high-risk patients with atrial fibrillation, as well as a reduction in its incidence.

In patients with chronic heart failure, Mohseni et al. [25] found that influenza vaccination is associated with a reduced risk of hospitalization, particularly for cardiovascular causes. Christiansen et al. [26] a cohort of intensive care unit survivors admitted for various medical and surgical conditions not necessarily related to cardiovascular disease was included. Their results showed that vaccinated patients had a lower risk of stroke and one-year mortality compared with unvaccinated ICU survivors. Lam et al. [27] demonstrated that, in older adults with a history of stroke, vaccination was associated with lower risks of post-stroke pneumonia, septicemia, urinary tract infection, and 30-day in-hospital mortality.

Wu et al. [28] evaluated influenza vaccination for secondary prevention of cardiovascular disease, showing significant reductions in all-cause mortality, myocardial infarction or cardiovascular death, and hospitalization for heart failure. Gotsman et al. [29] reported that vaccination in patients with heart failure is associated with improved clinical prognosis, including greater survival and fewer deaths and hospitalizations. Pang et al. [30] showed that vaccination reduces hospitalizations in older adults with cardiovascular or respiratory diseases; additionally, in a cardiovascular subtype analysis, they observed a 15% reduction in in-hospital mortality—which was more pronounced among patients with stroke—as well as a 6% reduction in the risk of recurrent hospitalization for ischemic heart disease [31].

Regarding dose comparison, Saade et al. [32] found that the high-dose vaccine does not provide additional protection against major cardiovascular events compared with the standard dose. Consistently, Christensen et al. [33] and NajafZadeh et al. [34] reported comparable effectiveness between both doses in terms of hospitalizations for pneumonia or influenza and all-cause mortality. Miró et al. [35] showed that, in patients with heart failure, seasonal influenza vaccination is associated with less severe decompensations and lower one-year mortality. Guo et al. [36] reported that vaccination may reduce the risk of major adverse cardiovascular events and acute coronary syndromes. Lei et al. [37] documented that, in critically ill patients with atrial fibrillation, vaccination is associated with improved survival. Finally, Yang et al. [38] observed that influenza vaccination is associated with a lower risk of ischemic stroke in older adult stroke survivors. Table 3 and Table 4 present in detail the methodological characteristics and main results of the included studies.

### 3.5. COVID-19 Vaccines

For the analysis of COVID-19 vaccines in adult patients with cardiovascular disease, five studies were included, comprising cohort designs, secondary cohort analyses, and self-controlled case series. The publication period ranged from 2022 to 2025, with follow-up intervals between 2019 and 2022. Overall, 128,158 vaccinated patients were included across the analyzed studies, with a mean age above 70 years.

The study by Akbar et al. [40] showed that COVID-19 vaccination was associated with a dose-dependent reduction in all-cause mortality, as well as lower rates of hospitalization and revascularization procedures. In contrast, Miró et al. [39] observed that COVID-19 vaccination in older adults with acute heart failure was associated with an increase in hospitalizations. Conversely, Johnson et al. [41] reported a significant reduction in all-cause hospitalization rates and mortality in vaccinated older adults with heart failure.

The study by Sindet-Pedersen et al. [42] evaluated the risk of adverse events following vaccination in two cohorts—patients with heart failure who were vaccinated and those who were not—and found that, in the vaccinated cohort, messenger RNA (mRNA)-based vaccines were not associated with an increased risk of heart failure worsening, myocarditis, venous thromboembolism, or all-cause mortality compared with the unvaccinated cohort. Similarly, Ye et al. [43] investigated the safety of COVID-19 vaccines and found no increased risk of hospitalization for heart failure, major adverse cardiovascular events, or all-cause hospitalization after administration of BNT162b2 or CoronaVac vaccines in older adults with heart failure. Table 5 and Table 6 provide detailed methodological characteristics and the main findings of the included studies.

## 4. Discussion

### 4.1. Respiratory Syncytial Virus Vaccines

Respiratory syncytial virus vaccines in older adults with cardiovascular disease are associated with a lower incidence of all-cause cardiorespiratory hospitalization and a reduction in stroke incidence compared with unvaccinated patients. Two studies derived from prespecified secondary analyses of the DAN-RSV trial were included, which introduces relevant methodological considerations, particularly regarding statistical power and outcome definitions. The study by Lassen et al. [19] demonstrated a lower incidence of all-cause cardiorespiratory hospitalization in the RSVpreF-vaccinated group, with a relative reduction of approximately 10%. However, although the direction of the effect estimates suggests a potential benefit in terms of cardiovascular hospitalizations and events such as stroke, these findings did not reach statistical significance. This may be explained by insufficient power to evaluate specific cardiovascular outcomes, as these were not primary endpoints of the trial [19]. Therefore, these results should be interpreted with caution and considered hypothesis-generating rather than definitive. Similarly, the study by Pareek et al. [20] reported a lower incidence of stroke in the RSVpreF-vaccinated group. This finding is biologically plausible, given that acute RSV infection has been associated with a transient increase in the risk of thrombotic events, including ischemic stroke [20]. However, the interpretation of this result also requires caution.

RSV is an important cause of acute respiratory infections during the fall and winter months and is the leading cause of lower respiratory tract infections in children [44]. However, RSV also significantly affects older adults and can lead to exacerbation of underlying diseases, hospitalization, and death [45]. RSV is identified in 6% to 11% of outpatient visits for respiratory tract infection in older adults and in 6% to 15% of hospitalized patients admitted to intensive care units; moreover, between 1% and 12% of all adults hospitalized with RSV respiratory tract infection die [46]. Epidemiologic studies estimate an RSV disease burden of 5.2 million cases, 470,000 hospitalizations, and 33,000 in-hospital deaths among adults aged ≥ 60 years in high-income countries [47]. These findings are likely related to deficient RSV F-specific T-cell responses in older adults, contributing to increased susceptibility to severe RSV disease [48].

Recent studies have shown that hospitalization due to RSV respiratory disease is complicated by cardiovascular events in 14% to 22% of adult patients, including worsening congestive heart failure, acute coronary syndrome, and arrhythmias. Furthermore, underlying cardiovascular disease is associated with hospitalization in 45% to 63% of adults with confirmed RSV infection [9]. Adults with RSV infection and underlying cardiovascular disease have a higher risk of experiencing an acute cardiac event compared with those without cardiovascular disease (33.0% vs. 8.5%) [7]. These outcomes may be explained by the fact that RSV infections can induce a hypercoagulable state and increased thrombotic risk, driven by higher levels of fibrinogen and thrombin and enhanced platelet binding [9]. From this perspective, RSV vaccination emerges as a potential preventive strategy to reduce cardiovascular risk; however, the specific protective mechanisms in patients with cardiovascular disease have not yet been fully elucidated.

Several clinical trials have demonstrated acceptable efficacy and safety of a single dose of the prefusion RSV F protein-based vaccine adjuvanted with AS01E for preventing acute respiratory infection, lower respiratory tract disease, and severe RSV-related illness in adults aged ≥ 60 years, regardless of viral subtype and the presence of comorbidities [49,50,51]. These findings are consistent with the results presented. Nevertheless, given the high vulnerability of this population, vaccination decisions should always be made using an individualized risk–benefit assessment framework. Overall, although these studies suggest a potential protective effect of RSV vaccination on cardiovascular outcomes, the available evidence remains limited and heterogeneous, both due to the secondary nature of the prespecified analyses and the variability in the outcomes assessed. This underscores the need for adequately powered clinical trials specifically designed to evaluate cardiovascular events as primary endpoints in this high-risk population.

### 4.2. Pneumonia Vaccines

Pneumococcal vaccines administered sequentially may exert a protective effect by reducing the risk of cardiovascular disease. A study by Tong et al. [21] demonstrated that dual sequential pneumococcal vaccination, defined as an immunization strategy that administers two different vaccines at separate time points to maximize protection, is associated with a lower risk of cardiovascular events. In particular, the sequential combination of PCV13 and PPSV23 appears to confer a greater preventive effect against pneumonia compared with the administration of each vaccine alone. This effect may be explained by a broader immune response against a greater number of serotypes when a sequential strategy is used, compared with the use of PCV13 or PPSV23 alone. Additionally, the reduction in pneumococcal infection episodes due to vaccination may attenuate the systemic inflammation induced by pneumonia, thereby contributing to a decreased incidence of cardiovascular events [21].

Pneumonia is an acute respiratory infection of major clinical relevance, with severity ranging from mild illness to life-threatening conditions across all age groups. Pneumococcal infections remain a significant cause of pneumonia and mortality in older adults and have been linked to an increased risk of acute cardiovascular events both during the infectious phase and in the post-infection period [52,53,54,55,56]. Systemic inflammation, platelet activation, and endothelial dysfunction induced by pneumococcal infection are considered key mechanisms in the precipitation of cardiovascular events [9]. In this context, pneumococcal vaccination has been shown to significantly reduce the incidence of invasive pneumococcal disease and all-cause mortality in older adults, with a favorable safety profile [57,58].

Older adults are at high risk of both pneumonia and cardiovascular disease; therefore, preventing both conditions could reduce two of the main sources of disease burden in this population. In this setting, vaccines targeting respiratory pathogens may reduce cardiovascular risk by attenuating the systemic inflammatory response triggered by respiratory infections [21].

Several epidemiological studies have demonstrated that, in patients with cardiovascular disease, influenza vaccination is associated with a reduction in cardiovascular mortality and the incidence of composite cardiovascular events [59]. A meta-analysis reported that vaccination with PPSV23 is associated with a reduced risk of cardiovascular events and acute myocardial infarction (AMI), with a greater effect observed in the older adult population [60]. Consistently, cohort studies have reinforced these findings, showing that sequential vaccination with PCV13 and PPSV23 is associated with an additional reduction in cardiovascular risk compared with single vaccination, an effect that appears to be mediated, at least in part, by a reduction in pneumonia episodes [21]. Overall, these findings are consistent with the results presented and support the potential cardiovascular benefit of pneumococcal vaccination, particularly when sequential strategies are used in high-risk populations.

### 4.3. Influenza Vaccines

Influenza vaccination in older adults with cardiovascular disease is consistently associated with improved cardiovascular clinical outcomes, reduced mortality, and a lower incidence of adverse events, constituting a safe and clinically relevant intervention in this high-risk population. Several studies have evaluated the impact of influenza vaccination on different cardiovascular outcomes in older adults, primarily including ischemic heart disease, stroke, heart failure, and atrial fibrillation. Overall, the evidence suggests a cardioprotective effect; however, important heterogeneity exists in study designs, populations, outcome definitions, and analytical approaches, which must be considered when interpreting the findings. In the context of ischemic heart disease, Hsu et al. [22] observed a reduction in the risk of acute myocardial infarction in vaccinated older adults, particularly in settings where there was a good match between vaccine strains and circulating viruses. Nevertheless, this effect may be influenced by seasonal variations and differences in antigenic composition across influenza seasons, introducing a potential source of confounding. Consistently, Chiang et al. [23] and Wu et al. [28] reported reductions in major adverse cardiovascular events, including myocardial infarction and cardiovascular mortality. These effects may be explained, at least in part, by the prevention of influenza infection, which has been associated with platelet activation, endothelial dysfunction, and a procoagulant state that promotes the destabilization of atherosclerotic plaques. Regarding stroke, multiple studies suggest a potential benefit of vaccination. Chiang et al. [23] and Yang et al. [38] reported a reduction in the incidence of ischemic stroke, whereas Liu et al. [24] demonstrated a dose–response protective effect against hemorrhagic stroke in patients with atrial fibrillation. However, these findings are not entirely consistent across studies, which may be attributable to differences in risk stratification, cohort age, and the handling of time-varying confounders. In addition, the underlying mechanisms are not fully elucidated, although prevention of systemic inflammatory and prothrombotic states induced by influenza infection has been proposed as a key pathway. In patients with heart failure, Mohseni et al. [25], Gotsman et al. [29], and Miró et al. [35] consistently reported an association between vaccination and reductions in hospitalizations, decompensations, and mortality. However, most of these studies are observational, including designs such as self-controlled case series, which limits causal inference and hinders the identification of direct pathophysiological mechanisms. Moreover, factors such as healthcare adherence and the so-called healthy user bias may partially account for the observed effects. In the context of atrial fibrillation, Liu et al. [24] and Lei et al. [37] reported benefits in terms of reduced cerebrovascular events and improved survival in critically ill patients. Nonetheless, evidence in this subgroup remains limited and is largely derived from observational studies, highlighting the need for targeted research in this population. Additionally, studies in high-cardiovascular-risk populations, such as those by Christiansen et al. [26] and Lam et al. [27], suggest further benefits in terms of reduced mortality and infectious complications. However, these findings should be interpreted with caution due to the potential for residual confounding, particularly related to socioeconomic factors, access to healthcare, and health behaviors, as vaccinated individuals tend to have greater adherence to preventive measures. Regarding dose comparisons, studies by Saade et al. [32], Christensen et al. [33], and NajafZadeh et al. [34] found no significant differences between high-dose and standard-dose influenza vaccines in terms of cardiovascular outcomes or mortality, suggesting that the observed benefit may be more closely related to vaccination itself rather than the magnitude of the immune response. However, insufficient statistical power to detect subtle differences between strategies cannot be ruled out.

Influenza, a highly contagious viral respiratory illness, disproportionately affects older individuals and those with chronic conditions. Its clinical spectrum is broad; however, severe cases often progress to pneumonia, acute respiratory distress syndrome, and multiorgan failure [61]. Influenza vaccination is one of the most widely used and studied respiratory vaccines worldwide [62]. It is estimated that more than 500 million doses are administered annually, making it the respiratory vaccine with the highest global coverage and extensive accumulated experience in terms of safety and effectiveness [62]. Its effectiveness may vary according to several factors, including the recipient’s age and immune status, the type of vaccine administered, the circulating influenza virus types, subtypes, and lineages, and the degree of antigenic match between circulating strains and those included in the vaccine [63]. In older adults, the relevance of influenza vaccination is supported by multiple pathophysiological and clinical factors [64].

Older adults, particularly those aged ≥ 65 years, are at increased risk due to immunosenescence, the presence of chronic comorbidities, and a reduced immune response to vaccination [61]. Aging is associated with immunosenescence, a process characterized by diminished immune responsiveness that increases susceptibility to severe infections and their complications [3,4]. In addition, this population has a higher prevalence of cardiovascular diseases, which may decompensate in the context of acute respiratory infections [7,8,65]. Epidemiological studies report mortality rates ranging from 2.9 to 44.0 per 100,000 individuals among those aged 65–74 years, and from 17.9 to 223.5 per 100,000 among those aged ≥ 75 years [66]. In this context, influenza vaccines have demonstrated high effectiveness in preventing severe infection-related outcomes [67,68].

The impact of influenza vaccination in older adults with cardiovascular disease is particularly relevant for optimizing coverage in this vulnerable population. Although the underlying biological mechanisms are not fully elucidated, meta-analytic evidence demonstrates cardioprotective effects, reflected in reductions in all-cause mortality, cardiovascular mortality, and the incidence of stroke [69]. A meta-analysis of randomized controlled trials reported that influenza vaccination is associated with a significant reduction in the risk of major adverse cardiovascular events, including a lower incidence of myocardial infarction and cardiovascular mortality [70]. Consistently, other studies have observed significant reductions in all-cause mortality, mortality, and major cardiovascular events in patients with cardiovascular disease [70]. Furthermore, a randomized clinical trial demonstrated that the high-dose influenza vaccine is superior to the standard-dose formulation in reducing hospitalizations due to cardiovascular, respiratory, and heart failure-related causes [71]. These findings are consistent with individual observational evidence in older adults with cardiovascular disease, reinforcing the role of vaccination as a key preventive strategy in this high-risk group.

Despite general consistency in the direction of effect, discrepancies persist across studies, likely explained by substantial methodological differences, including heterogeneity in study designs (retrospective cohorts, observational studies, randomized trials), outcome definitions (composite vs. specific), inclusion of primary versus secondary prevention populations, and approaches to confounding adjustment. These limitations hinder direct comparability of results and underscore the need for cautious interpretation.

Overall, the available evidence supports a potential cardioprotective effect of influenza vaccination in older adults with cardiovascular disease. However, the inherent limitations of the included studies, along with variability in their findings, highlight the need for randomized controlled trials and well-designed prospective studies specifically aimed at evaluating cardiovascular outcomes as primary endpoints.

### 4.4. COVID-19 Vaccines

The available evidence indicates that COVID-19 vaccination in older adults with cardiovascular disease demonstrates an overall favorable profile of clinical benefit and safety, reflected in reductions in mortality, hospitalizations, and revascularization procedures, without a significant increase in major adverse cardiovascular events associated with mRNA-based or inactivated vaccine platforms. However, the heterogeneity of results—particularly in subgroups such as patients with acute heart failure—highlights the need for individualized assessment and further research to more precisely define the clinical impact in specific high cardiovascular risk settings. The study by Akbar et al. [40] demonstrated that COVID-19 vaccination is associated with a dose-dependent reduction in all-cause mortality, as well as lower rates of hospitalization and revascularization procedures. Mechanistically, this dose–response gradient may be explained by two complementary processes: first, a reduction in SARS-CoV-2 infections following vaccination, which have been linked to endothelial dysfunction, vascular inflammation, and atherosclerotic plaque instability; and second, a potential attenuation of systemic inflammation, given that mRNA vaccination has been shown to reduce inflammatory biomarkers such as interleukin-6 and C-reactive protein by approximately 35–40% in high-risk populations, with effects comparable to those observed with lipid-lowering therapies such as statins. Consistent with these findings, the study also reported a 12% reduction in the need for percutaneous coronary intervention and a 19% reduction in coronary artery bypass grafting, reinforcing a potential protective effect at the atherothrombotic level [40]. In patients with heart failure, findings show some degree of heterogeneity across studies. Johnson et al. [41] reported a significant reduction in all-cause hospitalization and mortality among vaccinated older adults, suggesting a clinically meaningful benefit in this high-risk population. Regarding safety, the studies by Sindet-Pedersen et al. [42] and Ye et al. [43] consistently found that COVID-19 vaccines, particularly mRNA-based vaccines, are not associated with an increased risk of heart failure worsening, myocarditis, venous thromboembolism, major adverse cardiovascular events, or all-cause mortality. These findings remain consistent despite methodological differences across studies, including observational designs and variations in outcome definitions. Although vaccination has traditionally been recommended for patients with heart failure when they are in a clinically stable condition, the current evidence supporting this recommendation remains limited and inconclusive. In fact, some data suggest that mRNA vaccination may be safe even in recently decompensated patients, without a significant increase in adverse events. In this context, important gaps in the literature persist, particularly regarding the optimal timing of vaccination, stratification by heart failure severity, and the differential impact according to vaccine type.

Coronavirus disease 2019, caused by SARS-CoV-2, has led to a major global pandemic over the past four years and is currently behaving as an endemic disease. With the ongoing emergence of new viral variants, COVID-19 remains a relevant public health threat despite the widespread availability of vaccines, which were rapidly approved worldwide to mitigate SARS-CoV-2 infection [72]. The cardiotropic mechanisms of SARS-CoV-2 and its exacerbating effects in older adults are largely related to extrapulmonary processes. Viral entry into cardiomyocytes and endothelial cells via the angiotensin-converting enzyme 2 receptor induces direct myocardial injury and vascular dysfunction, while the systemic inflammatory response promotes a prothrombotic state [73,74]. Additionally, hypoxemia and increased hemodynamic stress favor myocardial ischemia and acute heart failure, along with autonomic and electrolyte disturbances that predispose to arrhythmias [75,76]. In later stages, persistent inflammation, endothelial dysfunction, and myocardial fibrosis contribute to an increased risk of heart failure, arrhythmias, and thromboembolic or cerebrovascular events [75].

Older adults represent a particularly vulnerable group with a high need for vaccine protection, although they may also have increased susceptibility to vaccine-related adverse events [77]. This phenomenon may be partially explained by age-related immunosenescence, in which humoral immunity tends to increase after vaccination, whereas cellular immune responses are often attenuated, potentially limiting the durability and breadth of vaccine-induced protection [78]. Several studies have suggested that COVID-19 vaccination significantly reduces all-cause mortality and disease severity in older adults, including those with underlying cardiovascular disease [40,79]. One study showed that the incidence of thrombotic and venous events, such as myocardial infarction and stroke, is lower following vaccination, and that the risk of cardiovascular complications after SARS-CoV-2 infection is substantially reduced in vaccinated individuals [80]. Additionally, a cohort study in older adults with ischemic heart disease or heart failure demonstrated that COVID-19 vaccination is associated with a dose-dependent reduction in mortality, as well as lower rates of hospitalization for heart failure and coronary revascularization procedures, without a significant increase in major adverse cardiovascular events [40]. It is important to note that the study by Miró et al. [39] found that COVID-19 vaccination was associated with increased hospitalization rates in patients with heart failure. Upon analyzing the study, the authors describe that one factor that may have influenced the differential effects of influenza and COVID-19 vaccines in patients with acute heart failure is that most of the population had been infected with SARS-CoV-2 by the end of 2022, nearly two years after the onset of the pandemic, whereas influenza infections had been very low during that period. This may have made patients more susceptible to influenza infection, while the risk of SARS-CoV-2 infection would have been clearly lower. In this context, the protection provided by the seasonal influenza vaccine would have been significantly greater than that of the seasonal COVID-19 vaccine. This perspective should be weighed against the consideration that COVID-19 strains evolved rapidly and that some vaccines may not have protected against emerging SARS-CoV-2 variants at the time their data were collected [39]. In this context, these favorable findings support the incorporation of complete vaccination schedules, including booster doses, as an essential component of secondary prevention strategies in older adults with pre-existing cardiovascular disease and high clinical risk [40].

It is important to highlight that the study by Miró et al. [39] reported an increase in hospitalization rates among patients with heart failure following COVID-19 vaccination. However, a more detailed analysis of their findings suggests that this result should be interpreted with caution and within its specific epidemiological context. The authors propose that one of the key factors that may explain the differential effects observed between influenza and COVID-19 vaccines in patients with acute heart failure is the pattern of prior viral exposure. By late 2022, a substantial proportion of the population had already been exposed to SARS-CoV-2, potentially conferring a certain degree of natural immunity. In contrast, influenza virus circulation remained markedly low during much of the pandemic, which may have increased patient susceptibility to influenza infection. In this context, the seasonal influenza vaccine may have provided a more evident clinical benefit compared to the COVID-19 vaccine, whose impact may have been attenuated by pre-existing immunity in the population. Additionally, other methodological and clinical factors may contribute to this apparent discrepancy. These include differences in study design, the potential presence of indication bias, and variations in outcome definitions, particularly with respect to hospitalizations. Furthermore, the rapid evolution and emergence of new SARS-CoV-2 variants, along with possible mismatches between circulating strains and those included in vaccines during the study period, may have influenced the observed effectiveness [39].

On the other hand, the concurrent finding of lower in-hospital mortality suggests that, despite the increase in hospitalizations, vaccination may be associated with reduced disease severity, thereby supporting its overall protective effect. Taken together, these findings underscore the complexity of interpreting vaccine effects in high-risk populations and reinforce the need to consider both epidemiological and methodological factors when assessing heterogeneity in the results.

### 4.5. Limitations

This study has several limitations that should be considered when interpreting its findings. First, although a comprehensive literature search was conducted across multiple databases, it was restricted to English-language publications; therefore, relevant studies published in other languages may have been excluded. Second, the marked methodological heterogeneity among the included studies, as well as the diversity of observational and experimental designs, limited the feasibility of conducting a robust quantitative analysis and deriving reliable and comparable statistical estimates. Third, detailed stratification by age groups (e.g., 60–69, 70–79, 80–89, and ≥90 years) was not possible, as many primary studies reported aggregated results or focused on specific populations, thereby precluding more precise comparative analyses. Fourth, the methodology and reporting framework inherent to scoping reviews do not support quantitative synthesis due to the substantial heterogeneity among studies. Fifth, a key limitation of this study is that several of the included studies enrolled mixed populations in which individuals with cardiovascular disease represented only a subset of the overall cohort. Although we attempted to extract and analyze data specifically for participants with cardiovascular disease whenever subgroup information was available, many studies did not report outcomes stratified by cardiovascular status. Consequently, some of the reported endpoints may include individuals without cardiovascular disease, which could limit the direct applicability of the findings to this target population. Additionally, as this is a scoping review, the objective was to map and synthesize the available evidence rather than to provide pooled effect estimates. Therefore, a more precise quantification of vaccine effects exclusively in patients with cardiovascular disease would require studies with consistent subgroup reporting or the conduct of meta-analyses focused on well-defined cardiovascular populations. Finally, several studies did not provide complete information on key clinical variables—such as comorbidities, functional status, nutritional status, or degree of frailty—which may significantly influence both the magnitude and quality of the immune response, particularly in older adults with cardiovascular disease.

### 4.6. Recommendations

Based on the findings of this scoping review, the development and implementation of large-scale clinical trials that explicitly include older adults with different types of cardiovascular disease are recommended, ensuring adequate representation of advanced age subgroups and patients with multimorbidity. These studies should standardize the assessment of efficacy, safety, and immunogenicity outcomes, while also incorporating relevant geriatric variables such as frailty, functional status, nutritional status, and immunosenescence, in addition to the evaluation, control, and monitoring of relevant biomarkers.

Furthermore, it is a priority to promote the development and updating of specific clinical practice guidelines that integrate the available evidence on the effects of respiratory vaccines in older adults with cardiovascular disease, with tailored recommendations according to cardiovascular and geriatric risk profiles. Additionally, public health strategies should be implemented to improve vaccine acceptance, access, and adherence in this population, as well as to evaluate their real-world impact on clinically relevant outcomes such as hospitalizations, cardiovascular events, and mortality. These actions could contribute to optimizing the prevention of respiratory infections and reducing the disease burden in one of the most vulnerable population groups.

## 5. Conclusions

Vaccination against respiratory viruses in older adults with cardiovascular disease is consistently associated with a favorable profile of clinical efficacy and safety, as evidenced by reductions in all-cause and cardiovascular mortality, lower incidence of hospitalizations, cerebrovascular events, and revascularization procedures, without a significant increase in serious adverse events attributable to the different vaccine platforms. These benefits are particularly relevant in a population characterized by high biological vulnerability, immunosenescence, and a substantial burden of comorbidities, in whom respiratory viral infections act as triggers for cardiovascular decompensation. The systematic implementation of complete vaccination schedules, including booster doses, should be considered a central strategy for both primary and secondary prevention in older adults with cardiovascular disease, with direct implications for public health policies and the optimization of comprehensive care in this high-risk population.

## Figures and Tables

**Table 1:** Characteristics of Respiratory Syncytial Virus vaccines in older adults.

Author/Year	Country	Design	Network	Period	Vaccine Type	Total Sample	Vaccinated Sample	Age (Mean/Median)	Female	Unvaccinated Sample	Age	Female	Outcomes	Follow-Up
Lassen et al. 2025 [19]	Denmark	Pre-specified secondary analysis of a trial	DAN-RSV	2024–2025	RSV Vaccine	28,662	14,377	71.8	5186	14,285	71.8	5038	Hospitalizations Mortality	1 year
Pareek et al. 2025 [20]	Denmark	Pre-specified secondary analysis of a trial	DAN-RSV	2024–2025	RSV Vaccine	131,276	65,642	71.5	21,268	65,634	69.2	21,265	Effectivity	1 year

MACE: Major adverse cardiovascular events; RSV: Respiratory syncytial virus.

**Table 2:** Effects of Respiratory Syncytial Virus vaccines in older adults.

Author/Year	Vaccinated Sample	Cardiovascular Disease	Efficacy All-Cause Mortality	Hospitalizations	Safety/ MACE	Conclusions
Lassen et al. 2025 [19]	14,377	AF: 10,126 (70.5%) IHD: 9746 (67.8%) HF: 2973 (20.7%)	VE: −56.5%	VE: −2.4%, AF VE: 1.8%, MI VE: −4.7%, HF	-	Lower hospitalizations rate in the vaccinated group
Pareek et al. 2025 [20]	65,642	AF MI HF Stroke	-	ARR: 2.35, AF ARR: −2.06, MI ARR: −2.06, HF ARR: 5.76, Stroke	VE: 9.3%, MACE	The effectiveness of the vaccines was similar to that of the controls

AF: Atrial fibrillation; ARR: Absolute risk reduction; HF: Heart failure; IHD: Ischemic heart disease; MACE: Major adverse cardiovascular events; MI: Myocardial infarct; VE: Vaccine effectiveness.

**Table 3:** Characteristics of Influenza Vaccines in older adults.

Author/Year	Country	Design	Network	Period	Vaccine Type	Total Sample	Vaccinated Sample	Age (Mean/Median)	Female	Unvaccinated Sample	Age	Female	Outcomes	Follow-Up
Hsu et al. 2016 [22]	Taiwan	Retrospective cohort	LHID 2005	2007–2008	Influenza vaccine	202,058	93,051	75.91	46,243	109,007	74.55	54,069	Risk of AMI	9 months
Chiang et al. 2017 [23]	Taiwan	Retrospective case–control	NHIRD	2000–2013	Influenza vaccine	160,726	Case: 29,046 Controls: 33,285	76.8	-	Case: 51,317 Controls: 47,078	76.8	-	MACE	-
Liu et al. 2017 [24]	Taiwan	Cohort	NHIRD	2005–2012	Influenza vaccine	6570	2547	74.33	1187	4023	72.79	1 913	Risk of HS	-
Mohseni et al. 2017 [25]	UK	Self-controlled case series	CPRD	1990–2013	Influenza vaccine	59,202	59,202	74.7	29,553	-	-	-	Hospitalizations	-
Christiansen et al. 2019 [26]	Denmark	Cohort	NHIRD	2005–2015	Influenza vaccine	31,108	11,866	-	5192	19,242	-	67,043	Hospitalization All-cause mortality	1 year
Lam et al. 2019 [27]	Taiwan	Cohort	NHIRD	2000–2009	Influenza vaccine	50,496	25,248	-	11,332	25,248	-	11,332	In-hospital mortality Hospitalizations	30 days
Wu et al. 2019 [28]	Taiwan	Retrospective PM-cohort	NHIRD	2000–2013	Seasonal influenza vaccine	8700	4350	76.3	1527	4350	76.2	1505	All-cause mortality Hospitalizations	1 year
Gotsman et al. 2020 [29]	Israel	Retrospective cohort	Clalit Health Services	2017–2018	Influenza vaccine	6435	4440	77	2056	1995	74	970	All-cause mortality Hospitalizations	1 year
Pang et al. 2021 [30]	China	Retrospective cohort	UEBMI	2013–2016	Influenza vaccine	139,506	17,655	74	-	121,851	72.9	-	In-hospital death	-
Pang et al. 2022 [31]	China	Retrospective cohort	UEBMI	2013–2019	Influenza vaccine	713,488	95,060	74.1	53,788	618,428	72.9	343,227	In-hospital death Hospitalizations	-
Saade et al. 2022 [32]	USA	Post hoc analysis	CMS	2013–2014	Influenza vaccine	49,175	49,175	83.8	35,674	-	-	-	Hospitalizations	-
Christensen et al. 2024 [33]	Denmark	Prespecified analysis of randomized clinical trial	DANFLU-1	2021–2022	Influenza vaccine	2540	2540	72.6	909	-	-	-	All-cause hospitalization Mortality	1 year
NajafZadeh et al. 2024 [34]	USA	Emulator Clinical Trial	Medicare claims data	2016–2019	Influenza vaccine	106,786	106,786	79.96	60,634	-	-	-	All-cause mortality Hospitalizations	-
Miró et al. 2023 [35]	Spain	Secondary analysis of cohort	EAHFE	2018–2019	Influenza vaccine	6147	1339	85	654	5008	84	2756	All-cause mortality Decompensations	1 year
Guo et al. 2025 [36]	USA	Target trial emulation	YRHCD	2020–2022	Influenza vaccine	339,976	169,988	72	90,414	169,988	72	91,138	MACE	2 years
Lei et al. 2025 [37]	China	Retrospective PM-cohort	MIMIC-IV	2008–2019	Influenza vaccine	9500	4758	75.44	-	4742	75.76	-	All-cause mortality	1 year
Yang et al. 2025 [38]	China	Retrospective cohort	RHIP	2021–2022	Influenza vaccine	76,747	31,729	75	15,326	45,018	76	21,851	Stroke risk	1 year
Miró et al. 2025 [39]	Spain	Secondary analysis of cohort	EAHFE	2022	Influenza vaccine	4243	1841	86	1039	2402	84	1359	All-cause mortality Decompensations	1 year

AMI: Acute myocardial infarction; HS: hemorrhagic stroke; MACE: Major adverse cardiovascular disease.

**Table 4:** Effects of Influenza Vaccines in older adults.

Author/Year	Vaccinated Sample	Cardiovascular Disease	Efficacy All-Cause Mortality	Hospitalizations	Severe Decompensations	Safety/MACE	Conclusions
Hsu et al. 2016 [22]	93,051	IHD: 15,904 (17.1%) MI: 313 (0.34%) HF: 3928 (4.2%) Hypertension: 41,371 (44.5%) IS: 6350 (6.8%)	-	-	-	HR = 0.681, AMI	Vaccination was associated with a reduced risk of AMI
Chiang et al. 2017 [23]	62,331	MI: 15,627 (25.1%) Stroke: 46,704 (74.9%)	-	-	-	aOR = 0.80, MI aOR = 0.80, Stroke	Vaccination is associated with a reduced risk of MACE
Liu et al. 2017 [24]	2547	AF: 2547 (100%) CHF: 1298 (50.96%) Hypertension: 1939 (76.13%)	-	-	-	aHR = 0.72, HS	Vaccination reduces the incidence of hemorrhagic stroke
Mohseni et al. 2017 [25]	59,202	MI: 41,502 (49.2%) HF: 59,202 (100%) Stroke: 14,522 (17.2%) Hypertension: 38,753 (46%)	-	Overall IRR = 0.73	-	-	Vaccination is associated with a lower risk of hospitalizations
Christiansen et al. 2019 [26]	11,866	AF/Flutter: 6234 (17.9%) MI: 4067 (11.7%) CHF: 4847 (13.9%) Stroke: 5080 (14.6%) Hypertension: 12,773 (36.6%)	1 year (aHR = 0.92)	1 year (aHR = 0.93, MI) 1 year (aHR = 0.98, HF) 1 year (aHR = 0.84, Stroke)	-	-	Vaccination was associated with a lower risk of stroke and mortality
Lam et al. 2019 [27]	25,248	HF: 276 (1.1%) Stroke: 25,248 (100%) IHD: 1017 (4.0%) Hypertension: 9735 (38.6%)	30 days (OR = 0.60)	30 days (OR = 0.91, ICU admission)	-	-	Vaccination associated with reduced post-stroke complications and mortality
Wu et al. 2019 [28]	4350	AF: 660 (15.17%) MI: 4350 (100%) HF: 2062 (47.4%) Hypertension: 3918 (90.07%)	1 year (HR = 0.82)	HR = 0.83, HF	-	-	Vaccination was associated with a reduced risk of CVD, all-cause mortality and hospitalizations
Gotsman et al. 2020 [29]	4440	AF: 1743 (39%) MI: 1904 (43%) HF: 4440 (100%) CHD: 2992 (67%) Stroke: 1053 (24%) Hypertension: 3745 (84%)	HR = 0.80	HR = 0.83, CVD	-	-	Vaccination was associated with a reduction in deaths and hospitalizations
Pang et al. 2021 [30]	17,655	NR	aOR = 0.55	-	-	-	Vaccination was associated with a lower risk of in-hospital death
Pang et al. 2022 [31]	95,060	IS: 95,060 (100%) IHD: 95,060 (100%)	aOR = 0.85	OR = 0.92, IHD OR = 1.04, IS	-	-	Vaccination was associated with a lower risk of in-hospital death in patients with fewer comorbidities
Saade et al. 2022 [32]	49,175	HF: 10,160 (20.7%) Stroke: 9813 (20%) Hypertension: 39,009 (79.3%)	-	HR = 0.92, MACE HR = 0.96, ACS HR = 0.96, HF HR = 0.84, Stroke	-	-	Similar reductions in hospitalizations were observed with both doses
Christensen et al. 2024 [33]	2540	AF: 822 (32.4%) IHD: 913 (35.9%) Hypertension: 961 (37.8%)	IRR = 0.51	IRR: 0.87, CVD	-	-	All-cause mortality declined in a dose–response pattern
NajafZadeh et al. 2024 [34]	106,786	AF: 63,986 (59.9%) AMI: 17,547 (16.4%) IS: 28,621 (26.8%) Hypertension: 105,315 (98.6%)	HR = 0.92	HR = 0.97	-	-	All-cause mortality and hospitalizations declined in a dose–response pattern
Miró et al. 2025 [35]	1841	AF: 993 (54%) CAD: 440 (23.9%) AHF: 1841 (100%) Hypertension: 1603 (87.1%)	90 days (HR = 0.831) 1 year (HR = 0.885)	OR = 0.746, HFD	OR = 0.926	-	Vaccination is associated with less severe decompensation and lower all-cause mortality
Guo et al. 2025 [36]	169,988	ACS: 14,019 (8.2%) HF: 18,441 (10.8%) Stroke: 38,919 (22.9%) Hypertension: 141,085 (83%)	-	-	-	1 year (IRR = 0.86, MACE) 1 year (IRR = 0.87, ACS)	Vaccination was associated with a reduction in MACE and ACS
Lei et al. 2025 [37]	4758	AF: 4758 (100%) MI: 1229 (22.89%) CHF: 2447 (45.57%) Hypertension: 4084 (76.05%)	1 year (HR = 0.83)	-	-	-	Vaccination was associated with a reduction in all-cause mortality
Yang et al. 2025 [38]	31,729	AF: 3262 (10.3%) CAD: 2486 (7.8%) Stroke: 31,729 (100%) Hypertension: 17,169 (54.1%)	-	-	-	sHR = 0.84, Stroke recurrence sHR = 0.75, HS sHR = 0.86, IS	Vaccination associated with reduced ischemic stroke risk
Miró et al. 2023 [39]	1339	AHF: 1339 (100%) HVD: 274 (31.5%)	90 days (HR = 0.885)	aOR = 0.823	aOR = 0.934	-	Vaccination is associated with less severe decompensations and fewer hospitalizations

ACS: Acute coronary syndrome; AHF: Acute heart failure; aHR: Adjusted Hazard Ratio; AF: Atrial fibrillation; AMI: Acute myocardial infarction; aOR: adjusted Odds ratio; CABG: Coronary artery bypass grafting; CAD: Coronary artery disease; CHD: Coronary heart disease; CV: Cardiovascular; CVD: Cardiovascular Disease; CHF: Congestive heart failure; HF: Heart failure; HFD: Heart failure decompensation; HR: Hazard Ratio; HS: Hemorrhagic stroke; HVD: Heart valve disease; ICU: Intensive Care Unit; IHD: Ischemic heart disease; IRR: Incidence rate ratio; IS: Ischemic stroke; MACE: Major adverse cardiovascular events; MI: Myocardial infarct; NR: Not reported; OR: Odds Ratio; sHR: subdistribution Hazard ratio.

**Table 5:** Characteristics of COVID-19 Vaccines in older adults.

Author/Year	Country	Design	Network	Period	Vaccine Type	Total Sample	Vaccinated Sample	Age (Mean/Median)	Female	Unvaccinated Sample	Age	Female	Outcomes	Follow-Up
Miró et al. 2025 [39]	Spain	Secondary analysis of cohort	EAHFE	2022	COVID-19 vaccine	4243	3139	85	1769	1104	85	629	All-cause mortality Decompensations	1 year
Akbar et al. 2025 [40]	USA	Retrospective PM-cohort	TriNetX US	2020–2022	COVID-19 vaccine	148,472	74,236	73.90	25,601	74,236	74.30	25,428	All-cause mortality	1–2 years
Johnson et al. 2022 [41]	USA	Retrospective cohort	-	2021–2022	COVID-19 vaccine	7094	3898	73.9	1877	3196	-	-	All-cause mortality Hospitalizations	-
Sindet-Pedersen et al. 2023 [42]	Denmark	Secondary analysis of cohort	-	2019–2021	COVID-19 vaccine	87,734	43,850	-	15,612	43,884	-	15,624	All-cause mortality Worsening Safety	-
Ye et al. 2023 [43]	China	Self-controlled case series	-	2021–2022	COVID-19 vaccine	8201	3035	-	1523	5166	-	2901	Hospitalizations MACE	-

MACE: Major adverse cardiovascular events.

**Table 6:** Effects of COVID-19 Vaccines in older adults.

Author/Year	Vaccinated Sample	Dose	Cardiovascular Disease	Efficacy All-Cause Mortality	Hospitalizations	Revascularization Rates	Safety/ MACE	Conclusions
Miró et al. 2025 [39]	3139	-	AF: 1663 (53%) CAD: 717 (22.8%) HF: 3139 (100%) Hypertension: 2713 (86.5%)	90 days (aHR = 0.829) 1 year (aHR = 0.91)	aOR = 1.215	-	-	Vaccination was associated with increased hospitalizations and lower in-hospital mortality
Akbar et al. 2025 [40]	74,236	1st: 28,500 2nd: 32,000 3rd: 13,736	CAD: 67,327 (90.70%) HF: 38,017 (51.12%) Hypertension: 57,591 (77.58%)	1 year (HR = 0.65, 3rd dose) 2 year (HR = 0.40, 3rd dose)	1 year (HR = 0.85, HF) 2 year (HR = 0.90, HF) 1 year (HR = 0.94, AF) 2 year (HR = 0.93, AF)	1 year: PCI (HR = 0.86, CAD) 2 year: PCI (HR = 0.87) 1 year: CABG (HR = 0.83) 2 year: CABG (HR = 0.80)	1 year (HR = 1.43, Myocarditis) 2 year (HR = 1.36, Myocarditis)	All-cause mortality declined in a dose–response pattern
Johnson et al. 2022 [41]	3898	1st: 3898 2nd: 3253 3rd: 1053	HF: 3898 (100%) Hypertension: 2529 (64.9%)	HR = 0.87, 1st dose HR = 0.36, 2nd dose	HR = 0.68	-	-	Vaccination was associated with a lower likelihood of all-cause hospitalizations and mortality
Sindet-Pedersen et al. 2023 [42]	43,850	-	AF: 19,401 (44.2%) HF: 43,850 (100%) AMI: 10,266 (23.4%) Stroke: 5498 (12.5%) IHD: 21,055 (48%) Hypertension: 37,712 (86%)	90 days (Standardized risk = 2.23%)	-	-	90 days (Standardized risk = 0.01%, Myocarditis)	Vaccination was associated with a slight reduction in mortality. It was not associated with worsening HF or an increased risk of myocarditis
Ye et al. 2023 [43]	3035	-	MI: 341 (11.2%) HF: 3035 (100%) IS: 40 (1.3%) Hypertension: 1791 (59%)	-	0–13 days (IRR = 0.60, HF) 14–27 days (IRR = 0.60, HF)	-	0–13 days (IRR = 0.19, MACE) 14–27 days (IRR = 0.10, MACE)	Hospitalizations and MACE declined in a dose–response pattern

aHR: Adjusted Hazard Ratio; AF: Atrial fibrillation; AMI: Acute myocardial infarction; aOR: adjusted Odds ratio; CABG: Coronary artery bypass grafting; CAD: Coronary artery disease; HF: Heart failure; HR: Hazard Ratio; IHD: Ischemic heart disease; IRR: Incidence rate ratio; IS: Ischemic stroke; MACE: Major adverse cardiovascular events; MI: Myocardial infarct; OR: Odds Ratio.

## Data Availability

Additional data related to this paper may be requested from the authors.

## Supplementary Materials

The following supporting information can be downloaded at: https://www.mdpi.com/article/10.3390/vaccines14040308/s1, Supplementary Materials S1, Design of Search Strategy.

## Abbreviations

The following abbreviations are used in this manuscript:

AMI	Acute myocardial infarction
CVD	Cardiovascular diseases
RSVpreF	Prefusion F protein-based RSV vaccine
RSV	Respiratory syncytial virus
SARS-CoV-2	Severe acute respiratory syndrome coronavirus 2
