# Supplementary material for: Effects of Respiratory Vaccines in Older Adults with Cardiovascular Diseases: A Scoping Review
Source: Vaccines (Basel). 2026 Mar 29;14(4):308. doi: 10.3390/vaccines14040308 (PMC13119681; doi:10.3390/vaccines14040308)
Supplement: Supplementary file 1 [file vaccines-14-00308-s001.zip › vaccines-4204525-supplementary.pdf]

Supplementary material S1

January 13, 2026

2016-2026

Design of Search Strategy

Databases: PubMed, Scopus, EMBASE and Web of Science platform.

PubMed (n= 627)

#1: (Influenza Vaccines[MeSH] OR “Influenza Vaccine*”[TIAB] OR “Influenza Virus Vaccine*”[TIAB] OR “Flu Vaccine*”[TIAB] OR “Trivalent Live Attenuated Influenza Vaccine”[TIAB] OR “LAIV Vaccine*”[TIAB] OR “Universal Influenza Vaccine*”[TIAB] OR “Universal Flu Vaccine*”[TIAB] OR “High Dose Trivalent Influenza Vaccine*”[TIAB] OR “Quadrivalent Influenza Vaccine*”[TIAB] OR “Trivalent Influenza Vaccine*”[TIAB] OR “Monovalent Influenza Vaccine*”[TIAB] OR Respiratory Syncytial Virus Vaccines[MeSH] OR “Respiratory Syncytial Virus Vaccine*”[TIAB] OR “RSV Vaccine*”[TIAB] OR Pneumococcal Vaccines[MeSH] OR “Pneumococcal Vaccine*”[TIAB] OR “Pnu Imune Vaccine*”[TIAB] OR “Pneumococcal Polysaccharide Vaccine*”[TIAB] OR Pneumovax[TIAB] OR COVID-19 Vaccines[MeSH] OR “COVID-19 Vaccine*”[TIAB] OR “SARS2 Vaccine*”[TIAB] OR “Coronavirus Disease 2019 Vaccine*”[TIAB] OR “2019-nCoV Vaccine*”[TIAB] OR “SARS-CoV-2 Vaccine*”[TIAB] OR “COVID-19 Virus Vaccine*”[TIAB] OR BNT162 Vaccine[MeSH] OR “BNT162 Vaccine*”[TIAB] OR “COVID-19 Vaccine Pfizer-BioNTech*”[TIAB] OR Tozinameran[TIAB] OR “BNT-162B2*”[TIAB] OR Comirnaty[MeSH] OR Comirnaty[TIAB] OR “Pfizer Covid-19 Vaccine*”[TIAB] OR ChAdOx1 nCoV-19[MeSH] OR “Oxford-AstraZeneca COVID Vaccine*”[TIAB] OR “ChAdOx1 COVID-19 Vaccine*”[TIAB] OR AZD1222[TIAB] OR Covishield[TIAB] OR 2019-nCoV Vaccine mRNA-1273[MeSH] OR “2019 nCoV Vaccine mRNA 1273*”[TIAB] OR “COVID-19 Vaccine Moderna*”[TIAB] OR mRNA-1273*[TIAB] OR “mRNA-1273.211*”[TIAB] OR Spikevax*[TIAB] OR Moderna COVID-19 Vaccine, Bivalent[MeSH]): 89, 500 results

#2: (Age-related immunosenescence[MeSH] OR “Age-related immunosenescence”[TIAB] OR Elderly[MeSH] OR Elderly*[TIAB] OR “Frail Elderly”[TIAB] OR “Frail Elders*”[TIAB] OR “Frail Older Adult*”[TIAB]): 3,910,852 results

#3: (Cardiovascular Disease[MeSH] OR “Cardiovascular Disease*”[TIAB] OR Heart Failure[MeSH] OR “Cardiac Failure”[TIAB] OR “Myocardial Failure”[TIAB] OR Coronary Artery Disease[MeSH] OR “Coronary Artery Disease*”[TIAB] OR “Coronary Arterioscleros*”[TIAB] OR Myocardial Infarction[MeSH] OR “Myocardial Infarction*”[TIAB] OR “Cardiovascular Stroke*”[TIAB] OR “Ischemic heart disease”[TIAB] OR Atrial Fibrillation[MeSH] OR “Atrial Fibrillation*”[TIAB] OR Stroke[MeSH] OR “Cerebrovascular Accident*”[TIAB]): 3,133,728 results

#4: #1 AND #2 AND #3: 627 results

Scopus (n= 687)

#1: TITLE-ABS-KEY((“Influenza Vaccine*” OR “Influenza Virus Vaccine*” OR “Flu Vaccine*” OR “Trivalent Live Attenuated Influenza Vaccine” OR “LAIV Vaccine*” OR “Universal Influenza Vaccine*” OR “Universal Flu Vaccine*” OR “High Dose Trivalent Influenza Vaccine*” OR “Quadrivalent Influenza Vaccine*” OR “Trivalent Influenza Vaccine*” OR “Monovalent Influenza Vaccine*” OR “Respiratory Syncytial Virus Vaccine*” OR “RSV Vaccine*” OR “Pneumococcal Vaccine*” OR “Pnu Imune Vaccine*” OR “Pneumococcal Polysaccharide Vaccine*” OR “Pneumovax” OR “COVID-19 Vaccine*” OR “SARS2 Vaccine*” OR “Coronavirus Disease 2019 Vaccine*” OR “2019-nCoV Vaccine*” OR “SARS-CoV-2 Vaccine*” OR “COVID-19 Virus Vaccine*” OR “BNT162 Vaccine*” OR “COVID-19 Vaccine Pfizer-BioNTech*” OR “Tozinameran” OR “BNT-162B2*” OR “Comirnaty” OR “Pfizer Covid-19 Vaccine*” OR “Oxford-AstraZeneca COVID Vaccine*” OR “ChAdOx1 COVID-19 Vaccine*” OR “AZD1222” OR “Covishield” OR “2019 nCoV Vaccine mRNA 1273*” OR “COVID-19 Vaccine Moderna*” OR mRNA-1273* OR “mRNA-1273.211*”)): 129,081 results

#2: TITLE-ABS-KEY((“Age-related immunosenescence” OR Elderly* OR “Frail Elderly” OR “Frail Elders*” OR “Frail Older Adult*”)): 1,077,366 results

#3: TITLE-ABS-KEY((“Cardiovascular Disease*” OR “Heart Failure” OR “Cardiac Failure” OR “Myocardial Failure” OR “Coronary Artery Disease*” OR “Coronary Arterioscleros*” OR “Myocardial Infarction*” OR “Cardiovascular Stroke*” OR “Ischemic heart disease” OR “Atrial Fibrillation*” OR “Cerebrovascular Accident*”)): 1,804,066 results

#4: #1 AND #2 AND #3: 687 results

EMBASE (n= 594)

#1: ('influenza vaccine*' OR 'influenza virus vaccine*' OR 'flu vaccine*' OR 'trivalent live attenuated influenza vaccine' OR 'laiv vaccine*' OR 'universal influenza vaccine*' OR 'universal flu vaccine*' OR 'high dose trivalent influenza vaccine*' OR 'quadrivalent influenza vaccine*' OR 'trivalent influenza vaccine*' OR 'monovalent influenza vaccine*' OR 'respiratory syncytial virus vaccine*' OR 'rsv vaccine*' OR 'pneumococcal vaccine*' OR 'pnu imune vaccine*' OR 'pneumococcal polysaccharide vaccine*' OR 'pneumovax'/exp OR 'pneumovax' OR 'covid-19 vaccine*' OR 'sars2 vaccine*' OR 'coronavirus disease 2019 vaccine*' OR '2019-ncov vaccine*' OR 'sars-cov-2 vaccine*' OR 'covid-19 virus vaccine*' OR 'bnt162 vaccine*' OR 'covid-19 vaccine pfizer-biontech*' OR 'tozinameran'/exp OR 'tozinameran' OR 'bnt-162b2*' OR 'comirnaty'/exp OR 'comirnaty' OR 'pfizer covid-19 vaccine*' OR 'oxford-astrazeneca covid vaccine*' OR 'chadox1 covid-19 vaccine*' OR 'azd1222'/exp OR 'azd1222' OR 'covishield'/exp OR 'covishield' OR '2019 ncov vaccine mrna 1273*' OR 'covid-19 vaccine moderna*' OR 'mrna 1273*' OR 'mrna-1273.211*'): 144,285 results

#2: ('age-related immunosenescence' OR elderly* OR 'frail elderly'/exp OR 'frail elderly' OR 'frail elders*' OR 'frail older adult*'): 864,468 results

#3: ('cardiovascular disease*' OR 'heart failure' OR 'cardiac failure' OR 'myocardial failure' OR 'coronary artery disease*' OR 'coronary arterioscleros*' OR 'myocardial infarction*' OR 'cardiovascular stroke*' OR 'ischemic heart disease' OR 'atrial fibrillation*' OR 'cerebrovascular accident*'): 2,372,498 results

#4: #1 AND #2 AND #3: 594 results

Web of Science (n= 95)

#1: (TS=("Influenza Vaccine*" OR "Influenza Virus Vaccine*" OR "Flu Vaccine*" OR "Trivalent Live Attenuated Influenza Vaccine" OR "LAIV Vaccine*" OR "Universal Influenza Vaccine*" OR "Universal Flu Vaccine*" OR "High Dose Trivalent Influenza Vaccine*" OR "Quadrivalent Influenza Vaccine*" OR "Trivalent Influenza Vaccine*" OR "Monovalent Influenza Vaccine*" OR "Respiratory Syncytial Virus Vaccine*" OR "RSV Vaccine*" OR "Pneumococcal Vaccine*" OR "Pnu Imune Vaccine*" OR "Pneumococcal Polysaccharide Vaccine*" OR "Pneumovax" OR "COVID-19 Vaccine*" OR "SARS2 Vaccine*" OR "Coronavirus Disease 2019 Vaccine*" OR "2019-nCoV Vaccine*" OR "SARS-CoV-2 Vaccine*" OR "COVID-19 Virus Vaccine*" OR "BNT162 Vaccine*" OR "COVID-19 Vaccine Pfizer-BioNTech*" OR "Tozinameran" OR "BNT-162B2*" OR "Comirnaty" OR "Pfizer Covid-19 Vaccine*" OR "Oxford-AstraZeneca COVID Vaccine*" OR "ChAdOx1 COVID-19 Vaccine*" OR "AZD1222" OR "Covishield" OR "2019 nCoV Vaccine mRNA 1273*" OR "COVID-19 Vaccine Moderna*" OR mRNA-1273* OR "mRNA-1273.211*")): 57,627 results

#2: (TS=("Age-related immunosenescence" OR elderly* OR "Frail Elderly" OR "Frail Elders*" OR "Frail Older Adult*")): 329,164 results

#3: (TS=("Cardiovascular Disease*" OR "Heart Failure" OR "Cardiac Failure" OR "Myocardial Failure" OR "Coronary Artery Disease*" OR "Coronary Arterioscleros*" OR "Myocardial Infarction*" OR "Cardiovascular Stroke*" OR "Ischemic heart disease" OR "Atrial Fibrillation*" OR "Cerebrovascular Accident*")): 999,505 results

#4: #1 AND #2 AND #3: 95 results
